# Novel Insights on Nutrient Management of Sarcopenia in Elderly

**DOI:** 10.1155/2015/524948

**Published:** 2015-01-29

**Authors:** Mariangela Rondanelli, Milena Faliva, Francesca Monteferrario, Gabriella Peroni, Erica Repaci, Francesca Allieri, Simone Perna

**Affiliations:** Department of Public Health, Experimental and Forensic Medicine, School of Medicine, University of Pavia and Endocrinology and Nutrition Unit, Azienda di Servizi alla Persona di Pavia, Via Emilia 12, 27100 Pavia, Italy

## Abstract

Sarcopenia is defined as a syndrome characterized by progressive and generalized loss of muscle mass and strength. The more rationale approach to delay the progression of sarcopenia is based on the combination of proper nutrition, possibly associated with the use of dietary supplements and a regular exercise program. We performed a narrative literature review to evaluate the till-now evidence regarding (1) the metabolic and nutritional correlates of sarcopenia; (2) the optimum diet therapy for the treatment of these abnormalities. This review included 67 eligible studies. In addition to the well recognized link between adequate intake of proteins/amino acids and sarcopenia, the recent literature underlines that in sarcopenic elderly subjects there is an unbalance in vitamin D synthesis and in omega-6/omega-3 PUFA ratio. Given the detrimental effect of these metabolic abnormalities, a change in the lifestyle must be the cornerstone in the treatment of sarcopenia. The optimum diet therapy for the sarcopenia treatment must aim at achieving specific metabolic goals, which must be reached through accession of the elderly to specific personalized dietary program aimed at achieving and/or maintaining muscle mass; increasing their intake of fish (4 times/week) or taking omega-3 PUFA supplements; taking vitamin D supplementation, if there are low serum levels.

## 1. Introduction

Sarcopenia is defined by the European Working Group on Sarcopenia in Older People (EWGSOP) [1] as a syndrome characterized by progressive and generalized loss of muscle mass and strength. Sarcopenia is a physiological phenomenon that usually starts in the fifth decade. van Kan (2009) has investigated the prevalence of sarcopenia in the population aged 60–70 years: in this age group, the prevalence ranged from 5 to 13% but increased to 11–50% in subjects aged >80 years [2].

Sarcopenia becomes responsible not only for the reduction of mobility and the level of autonomy of the elderly, but also for their ability to maintain good health. The functional reduction of the quadriceps muscle predisposes to a limitation in walking, with risk of falls and fractures of the femoral neck. A survey conducted in the USA has estimated the cost-related health consequences of sarcopenia to be 20–30 billion dollars [3].

In most elderly patients, the onset of sarcopenia is multifactorial. Like in all body tissues, muscle proteins are subjected to a constant process of synthesis and degradation; in healthy adults (with an adequate protein intake) this turnover is in balance, allowing the maintenance of a positive nitrogen balance and a constant muscle mass [4, 5].

In elderly, one of the pathogenic mechanisms leading to sarcopenia is altered muscle protein metabolism: the proteolytic processes are not accompanied by an adequate protein synthesis within the physiological turnover, and muscle cells lose progressively the sensitivity to the anabolic stimulus induced from the essential leucine and IGF-1 (insulin-like growth factor), thus manifesting the so-called “anabolic resistance” [6].

This phenomenon may be associated with other hormonal, functional, and nutritional factors, each of which may contribute to a greater or lesser extent—depending on gender, age, and clinical condition of the patient—to the progression of disease, defined as secondary sarcopenia [7]. With respect to nutritional causes, about 40% of subjects >70 years do not assume the current RDA (Recommended Dietary Allowances) of proteins (0.8 g/kg/day) [8]. The phenomenon depends upon several factors, each with a variable contribution: these include odontostomatological problems, capable of altering the masticatory function (therefore influencing the choice of foods with reduced content of proteins); a reduced capacity of digestion and assimilation of proteins in enteric tube; delayed gastric emptying, associated with a reduced gallbladder contractility and higher serum levels of the hormone cholecystokinin (CCK) and neuropeptide Y (PYY) (facilitating a long-lasting satiety [9]); a higher blood concentration of leptin in the elderly (showing that the anorexigenic signal prevails over the orexigenic one [10]); a propensity, increasing with age, to take sweet foods, easily chewable and already ready to eat, but not always adequate in the amino acid content.

In addition, costs are an issue, since a greater adherence to the Mediterranean diet is inversely related to BMI but leads to higher cost, which in 2006 were estimated at 1.2 €/day [11], due to the higher cost of meat and fish, compared with carbohydrates. Lastly, it is necessary to add that, according to available evidence, current RDA that define the protein intake in the elderly population should be revised, because for a number of reasons—many of which have already been discussed—they are often inadequate in terms of quantity and quality [12–15].

The FAO and the WHO indicate that an intake of 0.75 grams of high quality protein per kilogram of body weight is safe and adequate; however, for elderly subjects, it has been proposed to increase this value to 1.25 g/kg/day in order to avoid sarcopenia [16]. It is also necessary to consider that elderly subjects frequently present subclinical nutritional deficits, in particular of vitamins and minerals useful for the muscular tropism, such as vitamin D [17].

The more rational approach to delay the progression of sarcopenia is based on the combination of proper nutrition, possibly associated with the use of supplements and/or foods for special medical purposes, and a regular exercise program. Alternative treatments which are based on administration of hormone preparations such as testosterone, GH, and estrogens are still not universally accepted and require further investigation [18, 19].  Table 1 lists the nutrients and drugs which have been shown to increase the mass and/or muscle strength in humans or animal models.  Table 2 summarizes the studies (prospective cohort studies or randomized controlled trials) performed in elderly subjects to investigate the optimum dietary supplementation, other than proteins/aminoacids, for the treatment of sarcopenia.

Given this background, the aim of the present narrative review is to summarize the state of the art according to the extant literature about two topics: (1) the correct intake of protein and amino acids, in particular branched chain amino acids (BCAAs) need for prevention and treatment of sarcopenia; (2) the correct intake of other nutrients, such as antioxidant, vitamin D, and long-chain omega-3 polyunsaturated fatty acids, or dietary supplements, such as beta-hydroxy-methylbutyrate and creatine, in need for prevention and treatment of sarcopenia.

## 2. Methods

**Figure 1:**
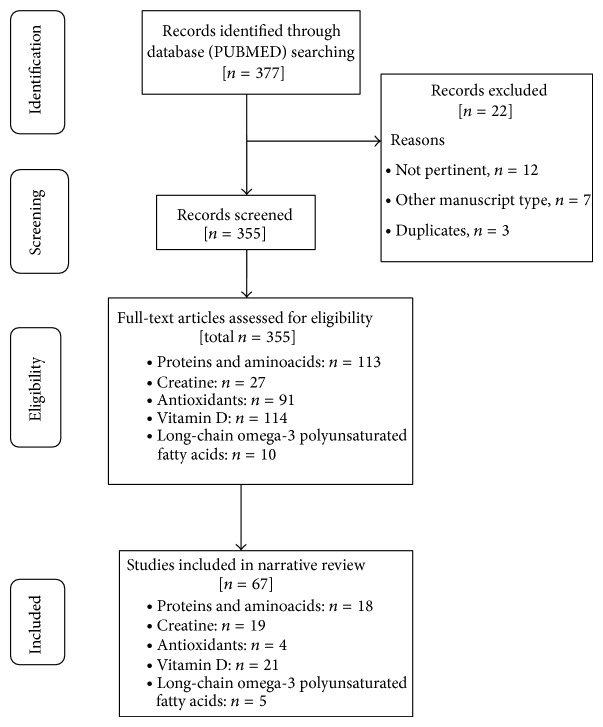
Flow diagram of narrative review of literature.

The present narrative review was performed following the steps by Egger et al. [20].  Table 3 showed the summary of methodology used. The step were (1) configuration of a working group: three operators skilled in endocrinology and clinical nutrition, of whom one acting as a methodological operator and two participating as clinical operators; (2) formulation of the revision question on the basis of considerations made in the abstract: “the state of the art on metabolic and nutritional correlates of sarcopenia and their nutritional treatment”; (3) identification of relevant studies: a research strategy was planned, on PUBMED (Public MedLine run by the National Center of Biotechnology Information (NCBI) of the National Library of Medicine of Bethesda (USA)), as shown in Table 3; (4) analysis and presentation of the outcomes: the data extrapolated from the revised studies were carried out in the form of a narrative review of the reports and were collocated in tables. The flow diagram of narrative review of the literature has been reported in Figure 1. At the beginning of each section, the keywords considered and the kind of studies chosen have been reported. Suitable for the narrative review were prospective cohort studies, randomized controlled trials (RCT), reviews, meta-analyses, cross sectional studies, and position paper which considered elderly with diagnosis of sarcopenia defined by the European Working Group on Sarcopenia in Older People (EWGSOP) [1].

## 3. Results

### 3.1. Aminoacids and Protein

This research has been carried out based on the keywords: “sarcopenia” AND “proteins” AND “aminoacids”; 113 articles were sourced. Among them, 1 observation study, 6 reviews, 2 cross sectional studies, 8 randomized controlled studies (RCT), and 1 position paper have been selected and discussed.

It is known that the amino acids, including branched chain amino acids (BCAAs), are necessary for the maintenance of muscle health in the elderly [21]. Approximately 300–600 grams of muscle proteins is degraded and resynthesized daily, with complete renewal of the pool of muscle protein in the human body occurring every 3-4 months. Food intake stimulates muscle protein synthesis, resulting in a positive protein balance. After taking a protein-rich meal, the degree of protein synthesis remains elevated for more than 5 hours, with a peak of 2-3 hours after the intake [22]. It has been shown that in adult subjects a dose of approximately 15–20 grams of protein (or 7.5 grams of essential amino acids) is sufficient to maximize the synthesis of muscle proteins [23].

Probably the elderly, compared with younger subjects, would require a larger amount of protein to obtain the same maximization of protein synthesis, probably 30 grams as shown by Pennings et al. [24]. The bioavailability of amino acids plays a major role in the regulation of protein metabolism in elderly subjects and therefore a nutritional therapy must necessarily aim also at the recovery of muscle and sensitivity to the stimulus induced by the protein-synthetic amino acids, in order to contrast anabolic resistance. Over the past few years, the analysis of the different nutritional strategies has allowed the definition of some key concepts, recently discussed in a position paper by the PROT-AGE Study group [16], which include the recommended amount of protein intake for the healthy elderly; the recommended amount of protein intake for the elderly with acute or chronic conditions; the role of physical activity in association with dietary intake to maintain muscle strength and function in elderly; the practical administration of food proteins (source and quality of dietary protein, protein intake timing, and energy intake).

#### 3.1.1. Recommended Amount of Protein Intake for Healthy Elderly

To maintain and recover the muscle, elderly subjects need to have a greater protein intake compared with younger subjects; older people should have an average intake of protein of 1.2/g/kg/day of body weight/day [16]. The threshold for anabolic meal intake of protein/amino acids must be greater in elderly subjects (i.e., 25 to 30 g of protein per meal, containing approximately 2.5–2.8 g of leucine), compared with young adults [24, 25]. The source of protein, timing of intake, and supplementation with amino acid supplements should be considered when making recommendations on the intake of dietary protein in the elderly.

#### 3.1.2. Recommendations in Protein Intake in the Course of Acute and Chronic Pathology

In subjects with pathological conditions, the amount of additional proteins to be taken or the protein requirement depends on the specific disease, its severity, nutritional status of the subject before the onset of the disease, and the impact of the disease on the state of nutrition. The majority of elderly patients who present an acute or chronic disease have an increased need for protein intake (1.2 to 1.5 g/kg body weight/day), while patients with critical illnesses or severe malnutrition have a need of protein equal to 2 g/kg body weight/day. Elderly subjects with severe renal impairment (estimated glomerular filtration rate < 30 mL/min/1.73 m^2^) who are not on dialysis are an exception and, conversely, must limit their protein intake.

#### 3.1.3. Quality of Protein and Specific Amino Acids

Not all dietary proteins have the same kinetic properties: the rate of absorption of dietary amino acids and their effect on the regulation of protein metabolism are dependent on the molecular characteristics of the protein. This characteristic gave rise to the distinction of dietary protein between fast and slow [26–28].

Previous work suggests that whey protein ingestion results in greater postprandial protein retention than does casein ingestion [29, 30].

The greater anabolic properties of whey than of casein are mainly attributed to the faster digestion and absorption kinetics of whey, which results in a greater increase in postprandial plasma amino acid availability and thereby further stimulates muscle protein synthesis [23, 26, 27, 31, 32].

Besides differences in protein digestion and absorption kinetics, whey and casein also markedly differ in their amino acid composition [26, 27, 32].

Whereas both proteins contain all the amino acids required to effectively stimulate muscle protein synthesis [33], whey has a considerably higher leucine content.

As regards differences between animal and vegetal sources, even if previous studies demonstrated that consumption of a meat-containing diet contributed to greater gains in fat-free mass and skeletal muscle mass with resistance training in older men than did a lactoovovegetarian diet [34, 35], more recent studies suggested that increases in muscle strength and size were not influenced by the predominant source of protein consumed by older men with adequate total protein intake [36].

#### 3.1.4. Branched Chain Amino Acids and Leucine

It has been suggested that leucine, which is an essential amino acid belonging to the category of the branched chain amino acids (BCAAs; valine, and together with the isoleucine, whose average requirement is 40 mg/kg/day), is critical to maintaining a healthy muscle tissue and liver. The main sources of leucine are chicken and fish, cottage cheese, lentils, sesame, and peanuts. Unlike many other amino acids, BCAAs are metabolized only in skeletal muscle, since the BCAA amino-transferase enzyme is not present in the liver, the site in which the enzymes metabolizing all other amino acids are present in maximum concentrations, and up to 58% of all the amino acids ingested (except BCAA) can be oxidized in the liver on the first pass. Skeletal muscle is able to oxidize only 6 amino acids during exercise: in addition to BCAAs, asparagine, aspartate, and glutamate. When combined with exercise training, BCAA supplementation increases testosterone and decreases cortisol to create an anabolic environment [37]. BCAAs represent 14%–18% of the total amino acid content of skeletal muscles [38]. At rest, BCAAs, in particular leucine, have an anabolic effect by increasing protein synthesis and/or a reducing the rate of protein degradation, resulting in a positive net muscle protein balance [39]. The infusion of BCAAs in humans elevates the phosphorylation and the activation of p70S6 kinase and 4E-BP1 in skeletal muscle [40]. Both p70S6 kinase and 4E-BP1 are downstream components of the mTOR signaling pathway, which controls RNA translation and synthesis of proteins, and which is recognized as the central node to support muscle hypertrophy [41]. Leucine is involved in the direct phosphorylation and activation of mTOR in skeletal muscle, further enhancing the protein synthetic response [42]. According to the WHO, for healthy people the daily demand of BCAA to cope with the normal loss in protein metabolism and turnover are the following: valine: 10 mg/kg body weight; isoleucine: 10 mg/kg body weight; leucine: 10 mg/kg body weight [43].

It was recognized that the leucine content of the meal is an important regulator of the synthesis of muscle proteins and influences body composition in the long term [44]. Further research has compared the intake of 10 grams of protein with 18% of leucine with a similar beverage containing 35% of leucine, concluding that the beverage with the highest concentration of leucine determines a greater signaling of protein synthesis, resulting in an inferior muscle catabolism by cortisol [45]. Other studies on leucine show that once the minimum requirement of leucine for protein synthesis is satisfied, leucine can be used to activate various signaling pathways, including mTOR. mTOR is a major regulator of protein synthesis, energy sensors, and sensors of nutrients and the availability of amino acids, particularly leucine. The mTOR pathway is activated when ATP levels are high and is blocked when ATP levels are reduced. The activation of mTOR is vital for skeletal muscle hypertrophy. Leucine presents significant activity to stimulate insulin synthesis, which may increase the availability of amino acids for the synthesis of muscle proteins; in addition, leucine inhibits the destruction of muscle proteins with consequent increased balance over time [46]. Although leucine is described as the most important of the three BCAAs, isoleucine and valine also play a role, although they have not shown the same potential as leucine. In fact, the hypertrophy induced by leucine decreases to zero as soon as the presence of the other two BCAAs is poor; regardless of the amount of leucine available to the muscles, muscle growth does not occur if the concentration of the other two BCAAs decreases below a given level [47].

#### 3.1.5. Beta-hydroxy-beta-methylbutyrate

Beta-hydroxy-beta-methylbutyrate (HMB) is a product of leucine metabolism that has been shown to slow protein breakdown in muscle tissue [48]. HMB may be effective at limiting the demands placed on the elderly subjects by acute stresses, such as sudden increases in physical activity, an immunologic challenge, or acute malnutrition [48, 49].

Daily supplementation of HMB (2 g/day), arginine, and lysine for 12 wk positively altered measurements of functionality, strength, fat-free mass, and protein synthesis, suggesting that the strategy of targeted nutrition has the ability to affect muscle health in elderly women [50].

In conclusion, an adequate intake of proteins (1.2/g/kg/day) is essential to prevent sarcopenia and aminoacids supplementation; in particular branched chain amino acids (leucine 2.5 g/day) as well as the intake of beta-hydroxy butyrate (2 g/day) is a well documented intervention for treating sarcopenia.

### 3.2. Creatine

This research has been carried out based on the keywords: “sarcopenia” AND “creatine”; 27 articles were sourced. Among them, 3 reviews, 11 RCT, 1 single blind study, 1 control case study, 2 observational studies, and 1 cross over study have been selected and discussed.

Creatine is chemically known as a nonprotein nitrogenous compounds; it is a tripeptide composed of three amino acids (glycine, arginine, and methionine). In the human body, creatine is synthesized in the liver and pancreas from the amino acids arginine, glycine, and methionine. Moreover, creatine is present in foods (meat and fish) and is taken with the diet in the amount of 1-2 grams per day. Approximately 95% of the creatine in the body is stored in skeletal muscles, as phosphocreatine (PCr) for about two-thirds of the total content, while the remaining part is stored as free creatine. The energy provided for the phosphorylation of adenosine diphosphate (ADP) to adenosine triphosphate (ATP) during and after intense exercise largely depends on the amount of PCr stored in the muscle. With the depletion of PCr during intense exercise, the availability of energy decreases due to the inability to resynthesize ATP in the amount required to keep the high-intensity exercise [51]. Age-associated reductions of creatine/phosphocreatine in skeletal muscle have been reported in some studies [52, 53], although not all studies agree [54, 55]. The reduction of muscle creatine is biologically plausible, due to aging and, possibly, to certain comorbidities, such as sarcopenia, and/or changes with age in behavior (reduced physical activity and/or changes in dietary behaviors, such as decreased intake of meat for edentulous). The type II muscle fibers have a higher content of phosphocreatine compared with type I fibers (86 against and 74 mmol/kg dm) [56], and sarcopenia is characterized by a preferential atrophy of type II fibers [57]. The progressive atrophy of type II fibers may therefore partly explain the reduced muscle creatine in the elderly. Moreover, the reduction of creatine in the muscle of the elderly is in line with previous evidence that documents an increased oxidative process in aged skeletal muscles, for example, with decreased dependence on glycolysis [58] and a decrease of lactate dehydrogenase [59]. Smith et al. (1998) first reported an increase in muscle PCr (30%) in middle-aged adults (58 years) as a result of short-term intake of high doses of creatine (0.3 g/kg/day for 5 days) [52]. In a similar study, Rawson et al. (2002) reported a smaller increase in muscle phosphocreatine (7 versus 35%) in older (70 years) compared to younger subjects (24 years), in response to the ingestion of creatine (20 g/day for 5 days) [55]. However, the muscle PCr baseline was greater in the young compared with adult subjects described by Smith et al. (1998) [52], while the elderly subjects described by Rawson et al. (2002) had greater initial muscle PCr compared with younger subjects [55]. Brose et al. (2003) reported an increase in total muscle creatine (30% men, 17% women) in elderly subjects (70 years) who underwent 14 weeks of resistance training associated with intake of creatine in a dose of 5 g/day [60], a result that is similar to the increases reported in younger adults [61, 62]. Eijnde et al. (2003) reported an increase in total muscle creatine (5%) and free creatine (21%) following 6 months of exercise program for muscular endurance associated with the creatine supplementation (5 g/day) [63]. From these studies, although limited in number, it seems that the muscle creatine in the elderly can be increased with oral creatine supplementation in a dose of 5 g/day, but that the magnitude of the response can be significantly affected by the initial muscle creatine. Wyss et al. (1998) have suggested that the increase in extracellular creatine may decrease the absorption of creatine muscle by decreasing the activity of creatinine [64]. Although Rawson et al. (2002) have reported the presence of increased creatine in the blood of elderly subjects (elderly 68.5 mol/l, young 34.9 mol/l) [55], Tarnopolsky et al. (2003) showed no decrease in the activity of creatine after creatine ingestion in older men and women [65]. The most peculiar discovery was an improvement in fatigue resistance, which has been shown in many different studies using different exercise test [52, 66–70]. Some investigators have reported an increase in strength [68, 69], but this has not always been demonstrated [66, 67]. Importantly, in subsequent publications, researchers have begun to evaluate the performance of activities of daily living (activity daily living, ADL) and have shown that creatine supplementation may improve the performance of daily tasks identified in the ADL scale [69, 71, 72]. The improvement of the performance of activities of daily living is an important finding, because of the association between the performance of ADL, fall risk, and mortality. Among the studies that have evaluated the muscle mass, the majority showed a greater increase in lean mass accretion after ingesting creatine in combination with resistance training [60, 73, 74]. A further advantage given by combining creatine supplementation with resistance exercise is the increase in bone mineral content. Chilibeck et al. (2005) showed a greater increase (3.2 versus 1%) of bone mineral content in older men (71 years) after 12 weeks of creatine supplementation (0.3 g/kg for 5 days, 0.07 g/kg for 11 weeks), in combination with training against resistance compared to training alone [75]. Dalbo et al. (2009) have stated that creatine is an effective intervention to combat sarcopenia [76]. The timing of creatine ingestion (i.e., 0.03–0.5 g/kg before and after the sessions of resistance training) can be more relevant than the amount of creatine. These novel findings have immediate application for research and health professionals for the design of optimal creatine application strategies for older individuals [51].

In conclusion, an adequate creatine supplementation could represent an intriguing intervention to counteract sarcopenia, in particular fatigue related to sarcopenia, although double-blind, placebo-controlled studies have not been conducted.

### 3.3. Antioxidants

This research has been carried out based on the keywords: “sarcopenia” AND “antioxidant”; 91 articles were sourced. Among them, 1 cross sectional study, 2 reviews, and 1 case control study have been selected and discussed.

Oxidative stress has been implicated as a central mechanism in the pathogenesis of sarcopenia [77].

Oxidative damage in skeletal muscle has been associated with the atrophy and loss of muscle function and fibers in sarcopenia [78].

Moreover, the accumulation of mitochondrial and nuclear DNA damage due to oxidative stress is thought to eventually compromise function, leading to the loss of myocytes [79].

Finally, reactive oxygen species can damage muscle tissue directly, but they also provide a trigger for the expression of inflammatory cytokines such as interleukin- (IL-) 1, tumor necrosis factor (TNF), and IL-6. In older age, a low-grade inflammatory state characterized by increased concentrations of inflammatory cytokines and acute phase proteins is common [80, 81].

Studies conducted among community-dwelling older adults suggest that the proinflammatory state does have a long-term consequence for sarcopenia. In the Longitudinal Aging Study Amsterdam, elevated IL-6 and CRP were associated with a loss of muscle strength over three years of follow-up [82].

Given this background, antioxidants (carotenoids, vitamin E, and vitamin C) should play an important role against sarcopenia.

Carotenoids inactivate free radicals and appear to modulate the transcription factors, such as the NFkB, which are involved in the regulation of IL-6 and other proinflammatory cytokines and have the ability, like alpha-tocopherol, to increase muscle strength [83, 84].

In the Women's Health and Aging Studies (WHAS) I and II, low serum carotenoid levels were associated with poor muscle strength [84]. Likewise in the InCHIANTI study, a low-carotene intake was associated with low physical performance [85]. These observations are consistent with a growing number of studies showing that a diet with high intake of fruits and vegetables is associated with a reduced risk of inflammation, hypertension, diabetes, cardiovascular disease, sarcopenia, and mortality [83].

Adherence to the Mediterranean diet, which is characterized by a high intake of fruits, vegetables, and whole grains, and lower consumption of red meat and saturated fats are associated with lower circulating IL-6 [86], and a recent trial showed the Mediterranean diet reduced IL-6 in adults [87].

In animal models, it was found that the ability of leucine to stimulate muscle protein synthesis is significantly decreased in aged rats compared to young adults. This defect was reversed when the animal was supplemented with antioxidants. The effects may be due to a reduction of the inflammatory state due to the antioxidants themselves [88]. In addition, the supplementation of vitamins E and C improves indices of oxidative stress associated with exercise in aged rats [89].

Concerning the specific vitamin E, in human studies, vitamin E has been shown to affect muscle strength of the elderly [90].

Several studies have shown the positive effects of vitamin E in reversing muscle damage during extensive muscle contraction (exercise) in healthy men. Vitamin E supplementation at a dose of 800 IU for 28 days resulted in lowering the expression of oxidative stress markers after a downhill run in both young and older men [91].

In another study, a longer supplementation period (12 weeks of vitamin E supplementation) lowered creatinine kinase level after exercise in young men, whereas older men showed decreased lipid peroxidation in both resting state and after exercise, indicating that vitamin E promotes adaptation against exercise induced-oxidative stress and reduced muscle damage [92].

In animal models, similar results were obtained [93, 94].

In conclusion, until today, despite the promising above-mentioned animal studies and studies on subjects without sarcopenia, no RCT studies have evaluated the efficacy of an integration with antioxidants in the elderly patient suffering from sarcopenia. These studies are needed, given that recent epidemiological studies in community-dwelling older adults show that low serum/plasma carotenoids are independently associated with low skeletal muscle strength and the development of walking disability.

### 3.4. Vitamin D

This research has been carried out based on the keywords: “sarcopenia” AND “vitamin D”; 114 articles were sourced. Among them, 6 RCT, 5 reviews, 5 observational studies, 4 longitudinal studies, 2 population studies, 2 prospective studies, and 2 cohort analytic studies have been selected and discussed.

Vitamin D deficiency is common among geriatric patients (2–60%) [95, 96]. Vitamin D is hydroxylated in the liver to 25 (OH) D. This step is still well presented in the elderly, but it can be affected by liver disease [97, 98]. Further hydroxylation occurs in the kidney with the formation of 1,25-(OH2) D; however the activity of hydroxylation by the kidney may decrease with age, in parallel with the decline in renal function [99]. Consequences are the following: a low level of vitamin D, renal failure, and a low intake of calcium may result in mild secondary hyperparathyroidism. Increased levels of parathyroid hormone (PTH) cause an increase in bone turnover that is associated with bone loss, predominantly cortical; secondary hyperparathyroidism has been proposed as the main mechanism through which vitamin D deficiency contributes to the pathogenesis of hip fracture [95]. The presence of receptors for vitamin D was demonstrated in many organs [100], and the active metabolite, 1,25-2(OH)D, has been shown to be implicated in numerous systems that reduce the cell growth and inducing differentiation [101, 102]. Epidemiological studies have suggested that vitamin D deficiency is associated with cancer of the colon and breast [103, 104]. Furthermore, the status of vitamin D influences the immune system and insulin secretion [105].

Many studies have shown that low levels of 1,25-(OH) D and 25-(OH) D are associated with lower muscle strength, increased body instability, falls, and disability in older subjects [106, 107]. A significant association between the genotypes of the receptor for vitamin D with the strength of the quadriceps was also observed [108]. In addition, studies on vitamin D supplementation in elderly subjects with vitamin D deficiency showed an improvement in physical function and isometric knee extension versus placebo [109, 110]. In parallel with the decline in muscle mass and function with aging, there is a reduction of the expression of the receptors VDR (vitamin D receptor) in skeletal muscle [111]. Previous research has linked some VDR polymorphisms with the reduction of muscle mass and function in the elderly [112], suggesting that vitamin D plays a role in the development and progression of sarcopenia [113]. Prospective analysis of LASA (the Longitudinal Aging Study Amsterdam) has shown that low levels of 25OHD are predictive of an increased risk of recurrent falls at 1 year [114], reduction of muscle mass and strength in 3 years [115], and admission to nursing homes in six years [116]. Limited data suggest that physical activity or hypertrophy of skeletal muscle may be an important source of vitamin D [113]. It has been shown that skeletal muscle is the main deposit of vitamin D in infant rats [117]. The outdoor exercise improves the levels of 25OHD in the elderly [118]. This suggests that physical activity may influence muscle hypertrophy independently of 25OHD. Pfeifer et al. (2002) suggested that the muscle building exercises can increase the levels of 25OHD [119], but further studies are needed [113]. Studies conducted to evaluate the effects of vitamin D supplements on functional abilities are partially contradictory. Some studies have shown that vitamin D intake did not improve physical performance [120, 121]. These studies were conducted in subjects with normal vitamin D. Conversely, studies conducted in 122 elderly subjects with low levels of vitamin D have been shown to benefit significantly from supplementation of vitamin D. In particular, Dhesi et al. [122] demonstrated in a group of subjects (mean age 77 years), history of falls and blood values of vitamin D less than or equal to 12 micrograms/liter of a daily supplementation with 600 UI ergo-calciferol induced a 3% improvement of physical performance, as assessed by the “Aggregated Functional Performance Time (AFPT)” [123], while in the control group a 9% deterioration was reported. With respect to the postural stability, which is related to the state of vitamin D [124, 125], the study showed 13% improvement, while the control group had a worsening equal to 3%. In terms of reaction time, the treated group had a 13% improvement compared with a deterioration of 3% of the placebo group. However, it was not demonstrated an improvement in muscle strength [126].

Recently, Muir and Montero-Odasso [127] performed a meta-analysis on a collection of studies that those aged over 60 years participate in randomized control trials of the effect of vitamin D supplementation without an exercise intervention on muscle strength, gait, and balance. The meta-analysis suggests that vitamin D supplementation (800–1000 IU) daily was associated with improvements of muscle strength and balance.

In conclusion, in vitamin D deficient sarcopenic subjects, dietary vitamin D supplementation (800–1000 IU daily) could be promising and interesting for treatment of sarcopenia.

### 3.5. Long-Chain Omega-3 Fatty Acids

This research has been carried out based on the keywords: “sarcopenia” AND “omega 3”; 10 articles were sourced. Among them, 2 RCT, 1 review, and 2 case control studies have been selected and discussed.

The ability of the skeletal musculature to use amino acids to build or renew constitutive proteins is gradually lost with age and this is partly due to a decline in skeletal muscle insulin sensitivity. Since long-chain omega-3 polyunsaturated fatty acids (LC*n*-3PUFA) from fish oil are known to improve insulin-mediated glucose metabolism in insulin-resistant states, some evidence in animal model suggests that polyunsaturated fatty acids might be a potentially useful therapeutic agent for the treatment and prevention of sarcopenia. It has been shown that providing feed enriched in fish oil to growing steers increases the activation (phosphorylation) of anabolic signaling proteins in muscle during administration of insulin and amino acids and increases the nonoxidative whole-body disposal of amino acids, an index of increased whole-body protein synthesis [128].

Furthermore, omega-3 fatty acid supplementation has been shown to prevent loss of muscle mass in burned guinea pigs [129].

In addition, omega-3 fatty acids present anti-inflammatory properties [130], which may also help alleviate the muscle anabolic resistance in older adults.

With respect to the effect of dietary omega-3 fatty acid supplementation on the rate of muscle protein synthesis and the anabolic signaling cascade in older adults, recently it has been demonstrated that omega-3 fatty acid supplementation had no effect on the basal rate of muscle protein synthesis but enhanced the hyperaminoacidemia-hyperinsulinemia-induced increase in the rate of muscle protein synthesis, which was accompanied by greater increases in muscle mTORSer2448 phosphorylation [131].

In conclusion, dietary LC*n*-3PUFA supplementation could potentially provide a safe, simple, and low-cost intervention to counteract anabolic resistance and sarcopenia.

## 4. Discussion

The more rational approach to delay the progression of sarcopenia is based on the combination of proper nutrition, possibly associated with the use of dietary supplements and a regular exercise program. An adequate intake of proteins (1.2/g/kg/day) is essential to prevent sarcopenia and aminoacids supplementation, in particular branched chain amino acids, is a well documented intervention for treating sarcopenia. Moreover, the current literature suggests that dietary LC*n*-3PUFA, vitamin D, and creatine supplementation could potentially provide a safe, simple, and low-cost intervention to counteract anabolic resistance and sarcopenia.

The author's nutritional recommendations have been showed in Table 4.

## Figures and Tables

**Table 1:** Nutrients and drugs that have been shown to present an activity of stimulation in increasing the mass and/or muscle strength in humans or in the animal model.

Nutrients	Proteins and amino acids (BCAAs) and creatine.
	Antioxidants (vitamin E, vitamin C, carotenoids, and resveratrol)
	Vitamins: vitamin D
	Long-chain omega-3 fatty acids

Drugs	Antagonists of mineral corticoids (Spironolactone)
	ACE inhibitors

Hormone replacement therapy	Testosterone (T)
	Growth hormone (GH)
	Combination therapy: T and GH
	Estrogen
	DHEA-S

**Table 2:** Studies (prospective cohort study or randomized controlled trial) performed in elderly subjects to investigate the optimum dietary supplementation, other than proteins, for the treatment of sarcopenia.

Nutrients	Author	Type of study	Results	Recommended treatment
Vitamin D	Snijder et al., 2006 [114]	Prospective cohort study	Poor vitamin D status is independently associated with an increased risk of falling in the elderly, particularly in those aged 65–75 yr.	
	Verhaar et al., 2000 [109]	Randomized controlled trial	Six months of alphacalcidol treatment led to a significant increase in the walking distance over 2 minutes.	Six months of vitamin D treatment (0.5 microg alphacalcidol)
	Gloth et al., 1995 [110]	Randomized controlled trial	In this cohort of homebound older people, improvement in vitamin D status was associated with functional improvement as measured by the Frail Elderly Functional Assessment questionnaire.	One month of therapy with either placebo or vitamin D (ergo-calciferol)

Beta-hydroxy-beta-methylbutyrate (HMB)	Flakoll et al., 2004 [50]	Randomized controlled trial	Daily supplementation of HMB, arginine, and lysine for 12 wk	Daily supplementation of HMB, arginine, and lysine for 12 wk positively altered measurements of functionality, strength, fat-free mass, and protein synthesis, suggesting that the strategy of targeted nutrition has the ability to affect muscle health in elderly women.

Long-chain omega-3 fatty acids	Smith et al., 2011 [131]	Randomized controlled trial	Omega-3 fatty acid supplementation had no effect on the basal rate of muscle protein synthesis but enhanced the hyperaminoacidemia-hyperinsulinemia-induced increase in the rate of muscle protein synthesis, which was accompanied by greater increases in muscle mTORSer2448 phosphorylation	1.86 g eicosapentaenoic acid (EPA, 20:5n23) and 1.50 g docosahexaenoic acid (DHA, 22:6n23), both as ethyl esters

**Table 3:** Summary of methodology.

Step	General activities	Specific activities
Step 1	Configuration of a working group	Three operators skilled in clinical nutrition: (i) one operator acting as a methodological operator (ii) two operators participating as clinical operators

Step 2	Formulation of the revision question	Evaluation of the state of the art on metabolic and nutritional correlates of sarcopenia and their nutritional treatment

Step 3	Identification of relevant studies on PUBMED	(a) Definition of the key words (sarcopenia, nutrients, and dietary supplement), allowing the definition of the interest field of the documents to be searched, grouped in inverted commas (“…”), and used separately or in combination; (b) use of the Boolean (a data type with only two possible values: true or false) AND operator that allows the establishments of logical relations among concepts; (c) research modalities: advanced search; (d) limits: time limits: papers published in the last 20 years; humans; languages: English; (e) manual search performed by the senior researchers experienced in clinical nutrition through the revision of reviews and individual articles on sarcopenia in elderly published in journals qualified in the Index Medicus

Step 4	Analysis and presentation of the outcomes	The data extrapolated from the revised studies were carried out in the form of a narrative review of the reports and were collocated in tables.

**Table 4:** Effect of nutrients or dietary supplementations on metabolic correlates of sarcopenia.

Nutrients or dietary supplementations	Recommendations	Specific effect
Proteins: average daily intake	It is recommended that the total protein intake should be 1–1.2 g/kg/day [16]	

Proteins: timing of intake	It is recommended to have 30 grams of protein of high biological value for each meal [25]	The elderly, compared with younger subjects, would require a larger amount of protein to obtain the same maximization of protein synthesis

Proteins: fast and slow	It is recommended to have whey protein ingestion because whey protein ingestion results in greater postprandial protein retention than does casein ingestion [31]	The greater anabolic properties of whey than of casein are mainly attributed to the faster digestion and absorption kinetics of whey, which results in a greater increase in postprandial plasma amino acid availability and thereby further stimulates muscle protein synthesis. Moreover, whey has a considerably higher leucine content

Proteins: animal and vegetal sources	When the total protein intake is adequate, the source of protein consumed (vegetal or animal) does not influence muscle strength and size [36]	Increases in muscle strength and size were not influenced by the predominant source of protein consumed by older men with adequate total protein intake

Branched chain amino acids (BCAAs),	It is recommended to have an adequate daily leucine supplementation (3 g/day)	A high proportion of leucine is required for optimal stimulation of the rate of muscle protein synthesis by essential amino acids in the elderly

Beta-hydroxy-methylbutyrate (HMB)	It is recommended to have a daily intake of beta-hydroxy butyrate (HMB-b, 2 g/day) because it can attenuate the loss of muscle mass and increase muscle mass and strength [50]	Beta-hydroxy-beta-methylbutyrate is a product of leucine metabolism that has been shown to slow protein breakdown in muscle tissue

Creatine	It is recommended to have an adequate creatine supplementation because it could represent an intriguing intervention to counteract sarcopenia and in particular fatigue associated with sarcopenia; the timing of creatine ingestion (i.e., 0.03–0.5 g/kg before and after the sessions of resistance training) can be more relevant than the amount of creatine [73, 76]	The ingestion of an adequate creatine supplementation determines the increase in muscle phosphocreatine (PCr) and the energy provided for the phosphorylation of adenosine diphosphate (ADP) to adenosine triphosphate (ATP) during and after intense exercise largely depends on the amount of PCr stored in the muscle

Vitamin D	It is recommended to have a dietary vitamin D supplementation (800–1000 UI ergo-calciferol/day) in vitamin D deficient sarcopenic subjects [127]	Dietary vitamin D supplementation determines an increase of the expression of the receptors VDR (vitamin D receptor) in skeletal muscle

Antioxidants. vitamin E, vitamin C, carotenoids, and resveratrol	It is recommended to have a diet with high intake of fruits, vegetables whole grains, which is rich in antioxidant, and lower consumption of red meat and saturated fats, because it is associated with a reduced risk of inflammation correlated to oxidative damage [83]	Adherence to the diet rich in antioxidants is associated with lower circulating IL-6

Long-chain omega-3 polyunsaturated fatty acids (LC*n*-3PUFA)	It is recommended to have dietary long-chain omega-3 polyunsaturated fatty acids (1.86 g eicosapentaenoic acid and 1.50 g docosahexaenoic acid/day) supplementation [131]	Long-chain omega-3 polyunsaturated fatty acids (LC*n*-3PUFA) supplementation improves insulin-mediated glucose metabolism in insulin-resistant states and increases the activation (phosphorylation) of anabolic signaling proteins in muscle during administration of insulin and amino acids and increases the nonoxidative whole-body disposal of amino acids, an index of increased whole-body protein synthesis
